# Size-tunable copper nanocluster aggregates and their application in hydrogen sulfide sensing on paper-based devices

**DOI:** 10.1038/srep24882

**Published:** 2016-04-26

**Authors:** Po-Cheng Chen, Yu-Chi Li, Jia-Yin Ma, Jia-Yu Huang, Chien-Fu Chen, Huan-Tsung Chang

**Affiliations:** 1 Department of Chemistry, National Taiwan University, Taipei 106, Taiwan; 2 Graduate Institute of Biomedical Engineering, National Chung Hsing University, Taichung 402, Taiwan

## Abstract

Polystyrene sulfonate (PSS), a strong polyelectrolyte, was used to prepare red photoluminescent PSS-penicillamine (PA) copper (Cu) nanoclusters (NC) aggregates, which displayed high selectivity and sensitivity to the detection of hydrogen sulfide (H_2_S). The size of the PSS-PA-Cu NC aggregates could be readily controlled from 5.5 μm to 173 nm using different concentrations of PSS, which enabled better dispersity and higher sensitivity towards H_2_S. PSS-PA-Cu NC aggregates provided rapid H_2_S detection by using the strong Cu-S interaction to quench NC photoluminescence as a sensing mechanism. As a result, a detection limit of 650 nM, which is lower than the maximum level permitted in drinking water by the World Health Organization, was achieved for the analysis of H_2_S in spring-water samples. Moreover, highly dispersed PSS-PA-Cu NC aggregates could be incorporated into a plate-format paper-based analytical device which enables ultra-low sample volumes (5 μL) and feature shorter analysis times (30 min) compared to conventional solution-based methods. The advantages of low reagent consumption, rapid result readout, limited equipment, and long-term storage make this platform sensitive and simple enough to use without specialized training in resource constrained settings.

Photoluminescent (PL) metal nanoclusters (NCs) have drawn considerable attention in the past decades due to their ultra-small size, which generates unique optical properties contrary to their bulk-metal counterparts. Based on these extraordinary properties, metal NCs have been used in sensors^1^,^2^,^3^,^4^, catalysts^5^,^6^, and imaging probes^7^,^8^,^9^. Although the exact PL mechanism of metal NCs remains unknown, it has been suggested that their luminescence highly depends on the size of the metal cores and surface ligands^10^,^11^. Various surface ligands such as small thiol molecules, polymers, and proteins have been utilized to synthesize metal NCs, demonstrating an important effect in determining the optical properties of NCs. For example, bovine serum albumin (BSA) protected gold (Au) NCs display near infrared emission and high biocompatibility^12^. However, the use of large quantities of BSA is costly. Alternatively, small thiol compounds such as 11-mercaptoundecanoic acid (11-MUA) are relatively inexpensive and can help tune the emission wavelength of Au NCs^13^ through reaction time and thiol ligand length. However, the low quantum yields (QYs) of small thiol protected Au NCs (typically < 1%) limits further applications.

Small thiol-protected Au NCs possessing aggregation-induced PL emission properties have been demonstrated by adjusting the organic solvent fraction^14^. These Au NCs were prepared in mixed solvents of ethanol and water and displayed QYs of up to 15% by increasing the ethanol fraction to 90%. Similar PL enhancement was also found in penicillamine protected PA-Cu NCs and glutathione protected Cu NCs^15^. In addition, pH-induced aggregation has also been used to induce emission of PA-Au-Cu NCs with a high QY (4.8%)^16^. Although metal aggregates have higher QYs than individual metal NCs, there are several drawbacks to this material, including poor solubility and low stability of aggregates in biological media. The organic solvents used to prepare the aggregates cause the material to have low biocompatibility. Ordinary sensitivity is also observed when these NCs are used in sensing devices due to severe hindrance of ligands, which prevent access to potential analytes. Although pH can be used to tune the degree of Cu NC aggregation, it is hard to precisely control this aggregation effect due to the fast protonation and deprotonation of surface ligands around their pKa values. Furthermore, irreversible PL quenching was observed when the environmental pH was increased, causing the Cu NCs to lose their sensitivity and selectivity towards potential analytes. To overcome these disadvantages, greater precision and control of metal NC aggregation without the use of organic solvents or pH is essential to enable both high QYs and good water dispersity for environmental or biomedical sensing applications.

Herein, we report a facile method to control the aggregation degree of PA-Cu NCs by adding different amounts of the negatively charged polyelectrolyte, PSS. 0.1 wt% PSS was used to obtain strong luminescence and outstanding dispersity of PSS-PA-Cu NCs in aqueous solution. The size, surface charge, and composition of PSS-PA-Cu NC aggregates were thoroughly examined by transmission electron microscopy (TEM), zeta potential, dynamic light scattering (DLS), and MALDI-TOF-MS.

The PSS-PA-Cu NC aggregates were used to detect H_2_S in spring-water samples to verify the chemical sensing capabilities of these new materials. H_2_S is a malodorous toxic gas that is produced in factory, mines, and the gas and oil industries^17^. It can lead to personal distress at low concentrations and may cause fatal consequences when the concentration is higher than 250 ppm^18^. Therefore, a portable and effective H_2_S sensor would be highly valuable for environmental monitoring. In this study, a method for H_2_S detection using PL nanomaterials was developed and demonstrated to exhibit high sensitivity, high selectivity, and short analysis time. Upon exposure of the PSS-PA-Cu NCs to H_2_S, the strong Cu-S interaction causes larger non-luminescent CuS particles to form, resulting in a PL quenching effect which is used for H_2_S sensing.

Moreover, to make the highly dispersed PSS-PA-Cu NC aggregates more practical for H_2_S detection, these materials were combined with a sensing platform composed of a microfluidic paper-based analytical device (μPAD)^4^,^19^,^20^,^21^,^22^,^23^. As a result, the analytical platform constructed using PSS-PA-Cu NC aggregates and μPADs allowed parallel and highly sensitive detection of H_2_S, featuring a detection limit of 650 nM in spring-water samples within a testing period of 30 min. Moreover, based on the PL quenching effect, H_2_S sensing could be distinguished using this device with a portable UV-light and the naked eyes.

## Results

### Synthesis of PSS-PA-Cu NC Aggregates

Cu NC aggregates can be prepared through charge neutralization and consequent aggregation of the copper thiolate complexes by either changing the inorganic solvent fraction or solution pH value^24^. Therefore, we hypothesized that adding polyelectrolytes to the solution and thus changing the electrostatic interaction between each NC could be used to finely control the aggregation degree of PA-Cu NCs. In order to verify this, various charged polyelectrolytes, including PSS, polyacrylic acid, polymethacrylic acid, polyethylene glycol, and polyethylene imine were introduced to the as-prepared PA-Cu NC aggregates. We observed that only PSS-PA-Cu NC aggregates remained well-dispersed in solution, while the other polyelectrolytes strongly precipitated the PA-Cu NCs (Fig. S1).

The outstanding stability of PSS-PA-Cu NCs over other polyelectrolytes can be attributed to the strong repulsion of surface PSS ligands, which have strong Coloumbic interactions (binding energy 821.8 kJ) with Cu^2+^ ions^25^. Sodium benzenesulfonate as a control failed to disperse the PA-Cu NC aggregates. The better stability of PSS-PA-Cu NC aggregates was attributed to the hydrophobic interaction between Cu-thiolate polymeric aggregates and the PSS backbone.

**Figure 1:**
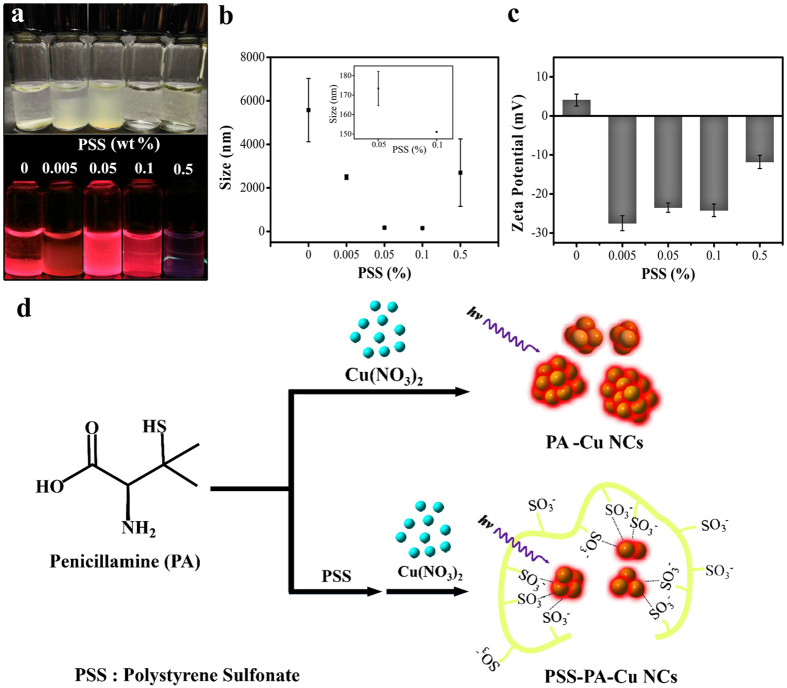
(**a**) Photographs of the PSS-PA-Cu NC aggregates synthesized under different concentrations of PSS, from 0.005–0.5 wt%. The upper row is shown under daylight and the bottom row is under UV illumination. (**b**) DLS analysis and (**c**) zeta-potential of PSS-PA-Cu NC aggregates in the presence of different concentrations of PSS from 0.005–0.5 wt%. (**d**) A scheme depicting a plausible mechanism for how PSS stabilizes PA-Cu NC aggregates.

To elucidate the outstanding stability of PSS-PA-Cu NC aggregates in aqueous media, the aggregates were prepared at different concentrations of PSS (0.005–0.5 wt%). As a control, PA-Cu NC aggregates were prepared in the absence of PSS. Figure 1a shows that the aggregation of the PSS-PA-Cu NCs decreases with increasing fraction of PSS. As-prepared PSS-PA-Cu NCs became fully dispersed when the PSS fraction exceeded 0.1 wt%. Under UV excitation, the PL intensity of PSS-PA-Cu NCs decreased with increasing PSS fraction, which implies that a higher fraction of PSS results in smaller PSS-PA-Cu NC aggregates. Although the mechanism of aggregation-induced emission of noble metal nanoclusters remains elusive, it is suggested that the existence of larger aggregates restricts the intermolecular vibration and rotation of nanoclusters to inhibit the non-radiative processes^14^.

The different PSS-PA-Cu NC aggregate samples were subjected to DLS analysis to understand how the concentration of PSS affected the aggregate size. As shown in Fig. 1b, the size of the PA-Cu NC aggregates decreased from 5.5 μm to 2.4 μm after 0.005 wt% PSS was introduced. When the concentration of PSS reached 0.1 wt%, the size of the PSS-PA-Cu NC aggregates significantly decreased to 152 nm, along with a decreased standard deviation of the aggregate size, indicating the formation of homogenous PSS-PA-Cu NC aggregates. The result confirmed our hypothesis that PSS effectively stabilizes PA-Cu NC aggregates. Higher PSS fractions (>0.1 wt%) did not form smaller PSS-PA-Cu NC aggregates, suggesting that the solution reaches a critical concentration of PSS (theoretically ~ 0.05 mM)^26^.

The surface charge of PSS-PA-Cu NC aggregates at various PSS concentrations was also investigated by measuring the solution’s zeta-potential. As shown in Fig. 1c, PA-Cu NC aggregates were slightly positively charged, while all PSS-PA-Cu NC aggregates were negatively charged due to the existence of strongly charged sulfonate groups on the PSS backbone. The scheme in Fig. 1d summarizes a plausible mechanism of formation for the PSS-PA-Cu NC aggregates. PSS-PA-Cu NCs prepared using 0.1 wt% PSS were characterized and used hereafter due to their high dispersity and strong luminescence.

### Characterization of PSS-PA-Cu NC Aggregates

The structure of PSS-PA-Cu NC aggregates was characterized using HR-TEM. The images of the aggregates (Fig. S2) appear vague due to the presence of large amounts of PSS moieties on the surface. Based on TEM observation, the average size of the individual Cu NCs in PSS-PA-Cu NC aggregates was estimated to be 1.7 nm (n = 100). The DLS results suggested the size of the PSS-PA-Cu NC aggregates was 152 nm. This discrepancy could be explained by our observation of small PA-Cu NCs in the TEM images, while DLS analysis revealed the overall aggregate structure.

MALDI-TOF-MS was used to analyze the composition of PSS-PA-Cu NC aggregates. As shown in Fig. S3, there is a broad band at 4120 Da indicating the existence of different sized species. To clarify the composition of PSS-PA-Cu NC aggregates, several control samples were investigated. PSS molecules revealed a broad band at 6000 Da, which implied PSS molecules carried more than 10 charges. In contrast, no signal was observed for PA-Cu NC aggregates (data not shown), which was attributed to the poor ionization efficiency of large aggregates. As a result, the *m/z* band observed at 4120 Da from the PSS-PA-Cu NCs sample appears to have originated from PA-Cu NC aggregates. Moreover, the composition of this band was assigned to Cu_25_PA_18_ according to the magic number model of noble metal NCs^10^,^27^. The tail band observed at 6047 Da for this same sample was assigned to PSS molecules. It was noted that intact PSS-PA-Cu NC aggregates were not identified due to fragmentation because of weak non-covalent bonding interactions between PSS and PA-Cu NCs. It is worth noting that using polyelectrolytes to disperse severe NC aggregates helps with MALDI-TOF-MS characterization. To the best of our knowledge, this is the first time that Cu NC aggregates were characterized using MALDI-TOF-MS. We believe this method provides an alternative approach to study severely aggregated NCs that have been prepared from pH- or solvent-induced aggregation methods.

### Optical Properties of PSS-PA-Cu NC Aggregates

**Figure 2:**
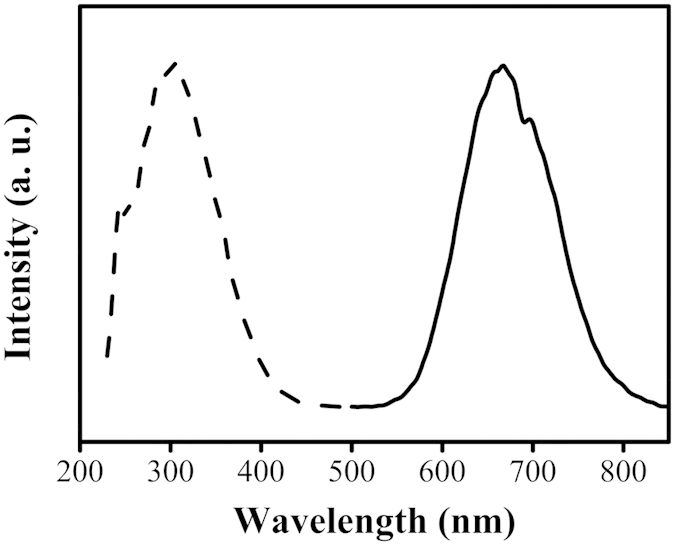
Excitation (black dotted line) and emission spectra (black solid line) of PSS-PA-Cu NCs. The excitation and emission wavelengths were 325 nm and 665 nm, respectively.

PSS-PA-Cu NC aggregates revealed the strongest emission band at 665 nm when excited at 325 nm (Fig. 2). The excitation spectrum of PSS-PA-Cu NCs exhibited a sharp peak located at 325 nm, which is in a good agreement with the material’s absorption spectrum (Fig. S4). The increased absorption value at regions > 300 nm implies the presence of ligand to metal charge transfer, which is commonly found in noble metal NCs^13^,^15^,^28^. The lack of an absorption band at visible wavelengths indicates that the size of the PSS-PA-Cu NC aggregates were too small to support plasmon resonance, which agrees with the average aggregate size measured using TEM. Using quinine sulphate as a standard (QY 54%), the QY of PSS-PA-Cu NC aggregates was determined to be 8%, which is comparable to previous reports for Cu NCs and PA-Au NC aggregates^29^,^30^.

The stability of PSS-PA-Cu NC PL was studied under various pH values and ionic strengths. Similar to previous reports on PA-Cu NCs and PA-Au-Cu NC aggregates, the luminescence of PSS-PA-Cu NC aggregates was observed to be highly sensitive to solution pH (Fig. S5). The luminescence of PSS-PA-Cu NCs dropped as pH was increased from 3.0 to 7.0 due to the reduction of inter/intra molecular hydrogen bonding of PA molecules. High ionic strength media also greatly impacted the PL of PSS-PA-Cu NC aggregates (Fig. S6). Their luminescence decreased 69% in the presence of 10 mM NaCl. In contrast, strong ionic strength media had little effect on PA-Cu NC aggregates (Fig. S7).

The differing response of PSS-PA-Cu NC and PA-Cu NC aggregates to salt may originate for two reasons. First, smaller-sized PSS-PA-Cu NCs are relatively easier for potential PL quenchers, such as oxygen, to access in the solution. Severely aggregated PA-Cu NCs would inhibit the effectiveness of these same quenching molecules. Second, the conformation of PSS is sensitive to salt due to the screening effects of intra- and inter-chain electrostatic interactions. The polyion chain becomes gradually coiled with increasing salt concentration, causing the decline of PSS viscosity. Under such conditions, the PSS can no longer stabilize PA-Cu NC aggregates, which causes PL quenching.

Adding highly viscous glycerol to the sample solution helped address the salt sensitivity of PSS-PA-Cu NC aggregates. When 50% glycerol was added, the PL of PSS-PA-Cu NC aggregates remained unchanged in the presence 50 mM NaCl (Fig. S8). The increased PL stability was attributed to the reduction of the screening effect of salt. As a result, 50% glycerol was added to all sensing solutions to increase the probes’ salt resistance in subsequent experiments.

**Figure 3:**
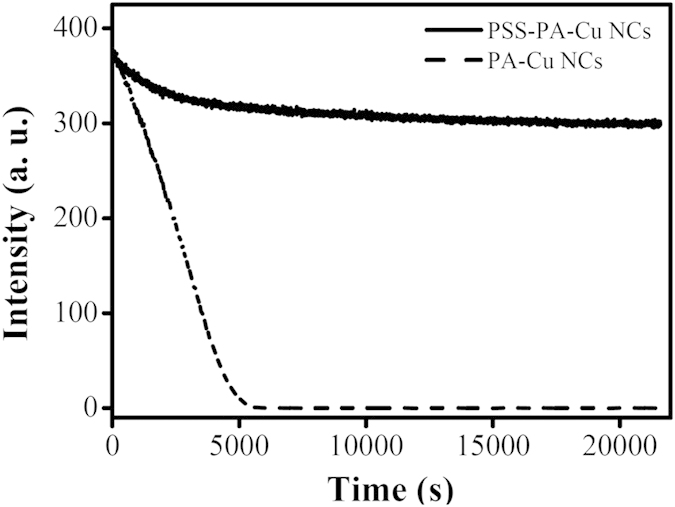
The photostability of PSS-PA-Cu NC aggregates (0.05×) and PA-Cu NC aggregates (0.05×) prepared in sodium phosphate buffer solutions (pH 3.0, 10 mM). The excitation wavelength was 325 nm.

The photostability of PSS-PA-Cu NCs was also better than PA-Cu NC aggregates, as shown in Fig. 3. After 6 h UV irradiation, the PL of PSS-PA-Cu NC aggregates decreased by less than 19%. In contrast, the PL of PA-Cu NC aggregates vanished after 1 h of UV exposure. The better photostability of PSS-PA-Cu NCs can be explained by the transfer of high-energy excited states from PA-Cu NC aggregates to surface PSS molecules. Therefore, the composition and conformation of PSS-PA-Cu NCs remains unchanged under UV irradiation. Similar phenomena have been observed in polymer coated semiconductor quantum dots^31^.

### Detection of H_2_S by PSS-PA-Cu NC Aggregates

Various techniques and platforms, such as chromatography, electrochemistry, titration, and transistor-based methods have been used for H_2_S detection^32^,^33^,^34^,^35^,^36^,^37^,^38^. However, these methods often require tedious sample pretreatment, expensive instrumentation, large quantities of sample, and/or well-trained personnel, all of which are problematic for practical applications. In contrast, PL is a promising method for sensing small molecules due to the short detection time, simple operation, and high spatial and temporal resolution^39^,^40^. For the detection of H_2_S, PL of PSS-PA-Cu NCs completely quenched within 1 min after addition of 20 μM H_2_S. The quenching mechanism was attributed to the formation of larger non-luminescent CuS particles, which were observed by TEM imaging (Fig. S9). The average diameter of individual PA-Cu NCs also increased (from 1.7 nm to 4.2 nm).

PA-Cu NC aggregates cannot be applied to H_2_S detection as the PL of these aggregates decreases by less than 16% in the presence of 20 μM H_2_S. The PL response towards H_2_S is also random (Fig. S10). The poor sensitivity of PA-Cu NC aggregates is due to the existence of large aggregates that inhibit the accessibility of H_2_S. Based on these results, we concluded that the dispersity of PA-Cu NC aggregates plays an important role for sensing performance.

**Figure 4:**
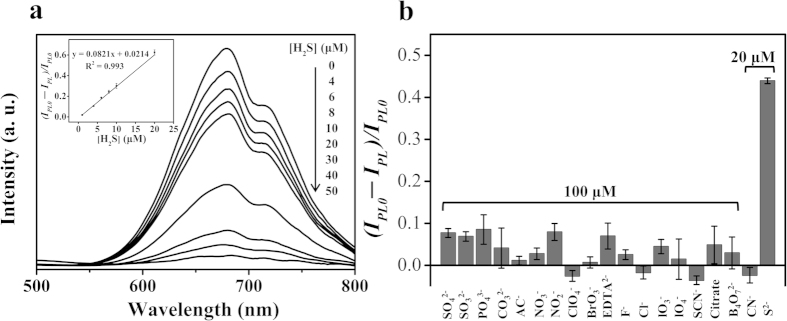
(**a**) PL spectra of PSS-PA-Cu NC aggregates (0.05×) as a function of H_2_S. Inset to (**a**): Linear plot [(I_PL0_ − I_PL_)/I_PL0_] of the PSS-PA-Cu NC aggregates against H_2_S concentration in sodium phosphate buffer solutions (10 mM, pH 3.0). (**b**) Selectivity of the PSS-PA-Cu NC aggregates (0.05×) toward H_2_S over other anions. The concentrations of S^2−^ and CN^−^ were 20 μM; other potential interference ions were at a concentration of 100 μM. Mixtures were prepared in sodium phosphate buffer solutions (10 mM, pH 3.0). I_PL0_ and I_PL_ are the PL intensities at 665 nm of the PSS-PA-Cu NC aggregates in the absence and presence of the potential interference ion, respectively.

The PL intensities of the PSS-PA-Cu NC aggregates in the absence and presence of H_2_S are denoted by *I*_PL0_ and *I*_PL_, respectively We observed that the relative PL intensity [(*I*_PL0_ − *I*_PL_)/*I*_PL0_] of the PSS-PA-Cu NC aggregates decreased with increasing concentration of H_2_S (Fig. 4a). A linear relationship (y = 0.082x + 0.021, R^2^ = 0.99) was found between the relative PL intensity and the concentration of H_2_S over the range of 1–20 μM. The limit of detection (LOD) of PSS-PA-Cu NC aggregates at a signal-to-noise ratio of 3 for H_2_S was determined to be 650 nM, which is much lower than the maximum level permitted in drinking water by the World Health Organization (15 μM; 500 ppb). This approach provided a comparable sensitivity to results reported by other H_2_S sensors^32^,^33^,^34^,^35^,^36^,^37^,^38^,^41^.

Moreover, PSS-PA-Cu NC aggregates also revealed outstanding selectivity for H_2_S. As shown in Fig. 4b, the PL intensity of PSS-PA-Cu NCs remained little changed in the presence of other potential interference ions (100 μM) that may be found in similar water sources (*e.g.* SO_4_^2−^, SO_3_^2−^, PO_4_^3−^, CO_3_^2−^, Ac^−^, NO_3_^−^, NO_2_^−^, ClO_4_^−^, BrO_3_^−^, EDTA^2−^, F^−^, Cl^−^, IO_3_^−^, IO_4_^−^, SCN^−^, citrate, B_4_O_7_^2−^, and CN^−^), while the PL intensity decreased by more than 50% in the presence of 20 μM H_2_S.

### Detection of H_2_S in Spring-Water Samples

To verify the viability of this probe for analysis of complicated environmental samples, PSS-PA-Cu NC aggregates were used for the detection of H_2_S in four spring-water samples. These water samples contained different interference ions and were of different pH values (1.6–7.5). The conventional methylene blue test method was used to determine the concentration of H_2_S in these spring-water samples in order to compare with and validate measurements made using our own NC probe. The results are summarized in Table 1. There was no significant difference found between the two parallel H_2_S testing methods, which suggests that the PSS-PA-Cu NC probes have real potential for environmental sensing. Moreover, compared to the methylene blue method, there was no need to add Fe^3+^ ions to catalyze the reaction in our sensing system, which makes our system simpler and more broadly applicable for use in environmental samples featuring wide pH ranges, as Fe^3+^ ions tend to form Fe(OH)_3_ and precipitate when pH is greater than 7.0^42^.

The main advantage of our PL-based method over absorption approaches, including the methylene blue method, is that PL-based sensing systems can analyze complicated samples with strong background color and turbidity.

### μPAD with PSS-PA-Cu NC Aggregates for H_2_S Detection

The conventional methylene blue test for H_2_S detection requires both an impinger and a spectrometer to aquire results. This method is relatively time consuming compared to the use of PSS-PA-Cu NCs for H_2_S sensing. In this study, μPADs were further incorporated to achieve low sample consumption, long-term storage and delivery, and rapid on-site environmental monitoring^43^,^44^.

**Figure 5:**
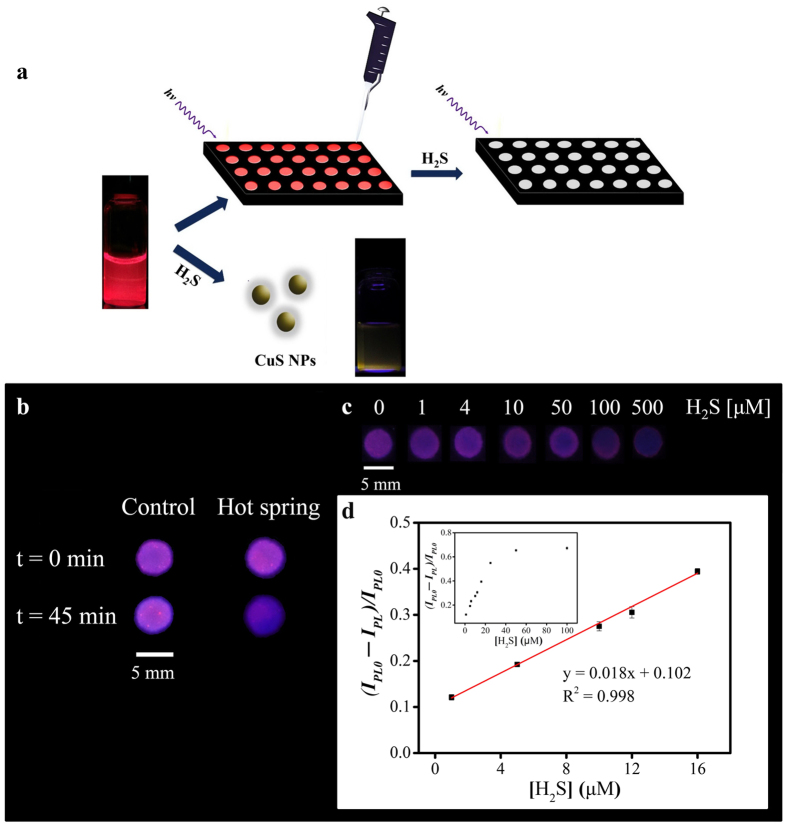
(**a**) A representative scheme for the detection of H_2_S on a PSS-PA-Cu NC/μPAD device. (**b**) Photographs of an on/off PSS-PA-Cu NC/μPAD for determination of the existence of H_2_S in Beitou hot spring-water samples for determination by the naked-eye. The control sample was phosphate buffer solution (pH 3.0, 10 mM) in the absence of H_2_S. (**c**) The upper row is the change in PL intensity of PSS-PA-Cu NC/μPADs at various H_2_S concentrations recorded by a smartphone. The lower row is the relative PL intensity plot [(I_PL0_ − I_PL_)/I_PL0_] of the PSS-PA-Cu NC/μPAD at 665 nm as a function of H_2_S. Inset: Linear plot [(I_PL0_ − I_PL_)/I_PL0_] of the PSS-PA-Cu NC aggregates against H_2_S concentration. The light source in (**b,c**) was a hand-held UV lamp.

Paper have been developed for biomedical sensing and analysis in resource-limited settings based on their advantages of low sample volume requirements, rapid detection, cost effectiveness, disposability, ease of fabrication, and wettability, which helps eliminate external flow control systems^19^,^20^,^21^,^22^,^23^. To study the performance of PSS-PA-Cu NC sensing on a paper device, the aggregates were spotted within a hydrophobic-wax-confined circle previously printed on cellulose paper^21^. This formed our nanomaterial-μPAD platform, used here for H_2_S detection. A representative scheme is shown in Fig. 5a.

The feasibility of this platform was first verified by the on/off detection of H_2_S from the Beitou spring-water sample. The prepared μPAD was placed overtop 20 mL tubes containing 15 mL spring-water sample. Due to the acidic environment of the spring sample (pH = 1.6), H_2_S solution evaporated into the atmosphere to quickly form H_2_S gas. The H_2_S gas caused the PL of the PSS-PA-Cu NC aggregates to quench on the μPAD. To accelerate this release of H_2_S gas, the spring-water sample was further heated to 70 °C. As shown in Fig. 5b, the luminescence of the platform decreased significantly after incubation with the spring-water sample at 70 °C for 45 min. In contrast, the PL of the PSS-PA-Cu NC aggregates in the presence of the control sample showed negligible change under the same conditions. It is worth noting that the results can be seen by the naked-eye, as well as facilely recorded and transmitted using a smartphone. Consequently, this analytical platform can be used as an on/off sensor for determination of H_2_S.

To further obtain the quasi-quantitative H_2_S concentration in the sample and demonstrate the ability of high-throughput and parallel analysis, various H_2_S aliquots (0–100 μM, 5 μL) were spotted onto the PSS-PA-Cu NC/μPAD device for preparation of a standard calibration curve. The change in the PL intensity was first recorded by a smartphone (Fig. 5d). The results show that the PL intensity decreased with increasing H_2_S concentration, from 0–100 μM. A calibration curve was constructed using a fluorescence microplate reader. A linear detection range was found from 2–10 μM (y = 0.0213x + 0.3531, R^2^ = 0.99). The LOD of the PSS-PA-Cu NC/μPAD device was 1 μM. The narrower linear range and poorer sensitivity (low slope) of the PSS-PA-Cu NC/μPAD device compared to the solution probe PSS-PA-Cu NC aggregates was attributed to the inhomogeneous reaction between the PSS-PA-Cu NC/μPAD device and the H_2_S solutions. However, the amount of each test mixture spotted on each hydrophobic-wax-confined circle was only 5 μL, which makes this H_2_S sensing platform faster, safer, and more cost-effective compared to a single PL analysis, which typically requires a sample volume of 1 mL. The usage of lower sample volume and direct interaction between the PSS-PA-Cu NC aggregates and H_2_S solution (rather than H_2_S gas) allows for more rapid detection of H_2_S (~30 min) compared to a single PL analysis (~60 min). Most importantly, PSS-PA-Cu NC/μPAD devices are ready to use and more suitable for long-term delivery and storage compared to PSS-PA-Cu NC aggregate solutions.

The quantitative analysis of PSS-PA-Cu NC/μPADs for H_2_S detection was further verified by determination of H_2_S concentration in Beitou spring-water samples. The water samples (5 μL) were spotted onto the μPADs and further analyzed using the microplate reader. One of the representative diluted samples shows that the relative PL intensity increased linearly (y = 0.0085x + 0.0697, R^2^ = 0.99) (Fig. S11) after spiking the Beitou spring-water solutions with various Na_2_S concentrations over the range of 1–20 μM. The concentration of H_2_S in the spring-water sample was determined to be 410 ± 16 μM (n = 3). A conventional methylene blue method was conducted on the same spring-water sample and determined that the H_2_S concentration was 414 ± 12 μM. Based on a t-test (the Student’s t value was 1.05 at 95% confidence level, three degrees of freedom), the two approaches did not provide significantly different results, indicating that our PSS-PA-Cu NC/μPAD platform holds great potential for practical environmental analysis.

## Discussion

This study shed light on understanding how to control the size of PA-Cu NC aggregates through the addition of different concentrations of PSS. Moreover, the composition of PA-Cu NC aggregates was determined for the first time using MALDI-TOF-MS due to the homogenous dispersion of PA-Cu NC aggregates in the presence of PSS. We believe that this strategy of adding highly charged polyelectrolytes could be used to determine the composition of other severely aggregated thiolate NCs.

The PSS-PA-Cu NC aggregates revealed excellent water dispersity, better photostability under UV irradiation, and remarkable sensitivity towards H_2_S (LOD: 650 nM) compared to PA-Cu NC aggregates. Moreover, the practicability of this probe was verified by determination of H_2_S in spring-water samples. In combination with μPAD technology, the portable hybrid platform allowed indirect on/off determination of H_2_S in the spring-water samples from released H_2_S gas. The advantages of this gas sensing method includes reduction of potential interferences in real water samples, while the conventional methylene blue method only allows direct determination of H_2_S in solution. Furthermore, PSS-PA-Cu NC/μPADs have the advantage of ultra-low sample volumes and shortened analysis time compared to conventional solution-based analysis. The sensing result can be obtained in 30 min compared to 60 min for a single PL analysis. In addition, complex optical instrumentation and manual processes that require well-trained technicians can be eliminated. Our results show that the PSS-PA-Cu NC/μPADs have great potential for monitoring H_2_S levels in gaseous samples. To broaden the practicability of this assay, multi-anion sensing arrays are under development using a combination of different nanomaterials and the μPAD platform.

## Methods

### Chemicals

Copper nitrate (Cu(NO_3_)_2_·3 H_2_O) was obtained from Showa (Tokyo, Japan). PA was purchased from Alfa Aesar (Lancs, UK). PSS (molecular weight ~70,000) and all other chemicals were purchased from Sigma-Aldrich (Milwaukee, WI). Ultrapure water (18.25 MΩ·cm) from a Milli-Q system (Millipore, Billerica, MA) was used to prepare all aqueous and organic-aqueous solutions. All chemicals and solvents were analytical grade and used without further purification.

### Preparation of PSS-PA-Cu NC Aggregates

In order to obtain PSS-PA-Cu NC aggregates for the detection of H_2_S, an aqueous solution of PA (0.1 M, 800 μL) was mixed with PSS (10 wt%, 10 μL) to form PSS-PA solution. Cu(NO_3_)_2_ was dissolved in 0.1 M nitric acid (0.1 M, 30 μL) and then rapidly added to the PSS-PA solution. When the color of the solution turned from brown to light yellow, the formation of PL PSS-PA-Cu NC aggregates was complete. To ensure reproducible results, the solution was shaken for an additional 2 h at 25 °C in the dark, followed by centrifugation at a relative centrifugal field of 16,000 (RPM) for 30 min. The collected supernatant was defined as a concentration of 1 × PSS-PA-Cu NC aggregates. The PSS-PA-Cu NC aggregates were stored in the dark at 4 °C before use.

### Preparation of μPADs

μPADs were built on Whatman grade 3 MM Chr cellulose chromatography cellulose paper (GE Healthcare; Little Chalfont, UK) using the solid-ink printing method. A pattern of wax was designed using Microsoft Office PowerPoint 2013 and printed using a Xerox solid ink printer (Color Qube 8570DN, Norwalk, Connecticut). The paper was then heated in an oven at 160 °C for 20 min to melt the solid wax and form hydrophobic sidewalls in a detection area defined by a 3 mm diameter circle.

### Detection of H_2_S Using PSS-PA-Cu NC Aggregates

The light-yellow PSS-PA-Cu NC aggregate solution (1×, 50 μL) was added to sodium phosphate buffer solutions (10 mM, pH 3.0, 950 μL) containing glycerol (50 wt%) and H_2_S to 1 mL, to give final H_2_S concentrations of 1–100 μM. After equilibration by slight shaking at room temperature for 1 h in the dark, the solutions were subjected to PL measurement.

### Detection of H_2_S Using PSS-PA-Cu NC Aggregates in Spring-Water Samples

Real sample tests were performed using spring water spiked with different concentrations of standard Na_2_S solutions. spring water samples were collected from three sources in Taiwan: Jinshan District; Beitou; Yangmingshan National Park of Taipei; and Su-ao of Ilan, Taiwan. All spring-water samples were filtered through 0.2 μm polyvinylidene fluoride membranes prior to testing. The water samples contained abundant ions including SO_4_^2−^, Cl^−^, Ca^2+^, and Na^+^ (pH 2.6). Aliquots (20 μL) of the spring-water samples were spiked with different standard of Na_2_S solutions (final concentrations of 1–50 μM). The H_2_S concentrations of the pretreated samples were then measured using the PSS-PA-Cu NC aggregates as described in the abovementioned analyses of the standard solutions. The concentrations of H_2_S in the samples were also determined by a standard methylene blue method, which has been approved by the American Public Health Association (APHA).^45^

### Detection of H_2_S on μPADs

PSS-PA-Cu NC aggregates were dissolved in ethanol in a 1:1 ratio to reduce the material’s surface tension for homogenous wetting on the cellulosic paper that makes up the μPAD sensors. 5 μL of this PSS-PA-Cu NC aggregate solution was then spotted on the hydrophilic printed circles and dried for 10 min. Different concentrations of H_2_S solutions, ranging from 1–20 μM, in sodium phosphate buffer (10 mM, pH 3.0, 5 μl) were added separately into the printed circles, followed by 30 min incubation in the dark. The PL intensity of this platform was then recorded using a smartphone (Apple iPhone 6, Cupertino, CA) and the fluorescence was determined using a microplate reader (BioTek Synergy H1, Winooski, VT).

### Detection of H_2_S in Spring-Water Samples on μPADs

Aliquots (20 μL) of spring-water samples from the Beitou district of Taipei were spiked with standard Na_2_S solutions (final concentrations of 1–15 μM) and diluted to 1 mL using sodium phosphate buffer (10 mM, pH 3.0). The pretreated samples were then spotted on the printed circles on the μPAD sensor where PSS-PA-Cu NCs aggregates had been previously loaded, followed by 30 min incubation in the dark. The PL intensity of this platform was recorded and determined using a smartphone and fluorescence microplate reader. In addition, the concentrations of H_2_S in the spring-water samples were also determined using a standard methylene blue method.

## Additional Information

**How to cite this article**: Chen, P.-C. *et al*. Size-tunable copper nanocluster aggregates and their application in hydrogen sulfide sensing on paper-based devices. *Sci. Rep.* **6**, 24882; doi: 10.1038/srep24882 (2016).

## Supplementary Material

Supplementary Information

## Figures and Tables

**Table 1:** H_2_S concentration in spring water samples determined by PSS-PA-Cu NC aggregates (n = 3).

Location	pH value	Qualified H_2_S by our method (μM)	Qualified H_2_S by methylene blue method (μM)
Beitou (Longnaitang hot spring)	1.6	900 ± 11	940 ± 17
Yangmingshan National Park (Lengshuikeng)	6.1	150 ± 10	170 ± 20
Jinshan District	2.6	642 ± 14	626 ± 15
Yilan (Suao Cold Spring)	7.5	–	–
