# Supplementary material for: Size-tunable copper nanocluster aggregates and their application in hydrogen sulfide sensing on paper-based devices
Source: Sci Rep. 2016 Apr 26;6:24882. doi: 10.1038/srep24882 (PMC4845058; doi:10.1038/srep24882)
Supplement: Supplementary Information [file srep24882-s1.pdf]

Electronic Supplementary Information for

Size-Tunable Copper Nanocluster Aggregates and Their Application in Hydrogen Sulfide Sensing on Paper-Based Devices

Po-Cheng Chen,[†] *Yu-Chi Li,*[†] *Jia-Ying Ma,*[†] *Chien-Fu Chen*[‡*] *and Huan-Tsung Chang*[†*]

[†]Department of Chemistry, National Taiwan University, Taipei 106, Taiwan

[‡]Graduate Institute of Biomedical Engineering, National Chung Hsing University, Taichung 402, Taiwan

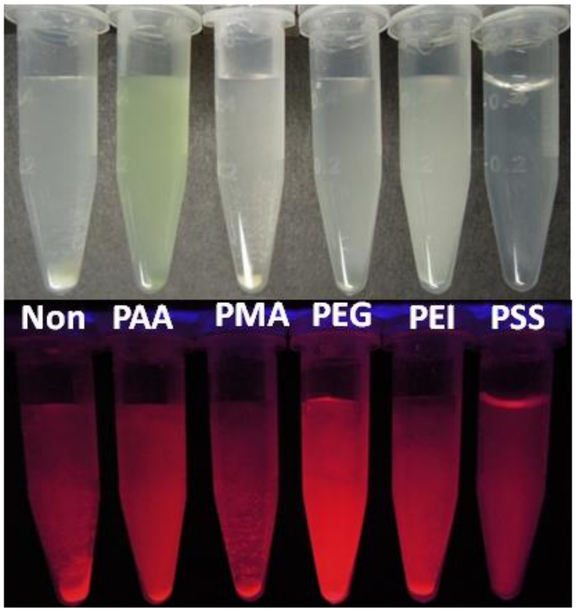

Figure S1. Photographs of PA-Cu NC aggregates (upper row: under daylight; bottom row: under UV light) prepared in the absence (Non) and presence of 0.1 wt% of various charged polyelectrolytes, including polyacrylic acid (PAA), polymethacrylic acid (PMA), polyethylene glycol (PEG), polyethylene imine (PEI), and PSS from left to right.

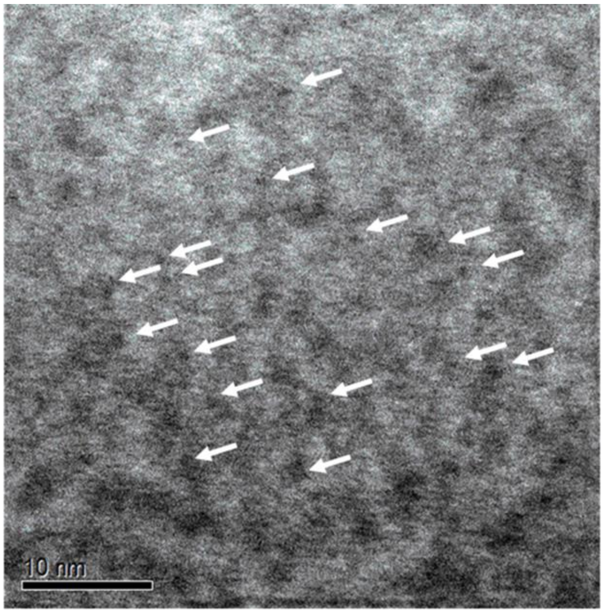

Figure S2. HR-TEM images of the PSS-PA-Cu NC aggregates.

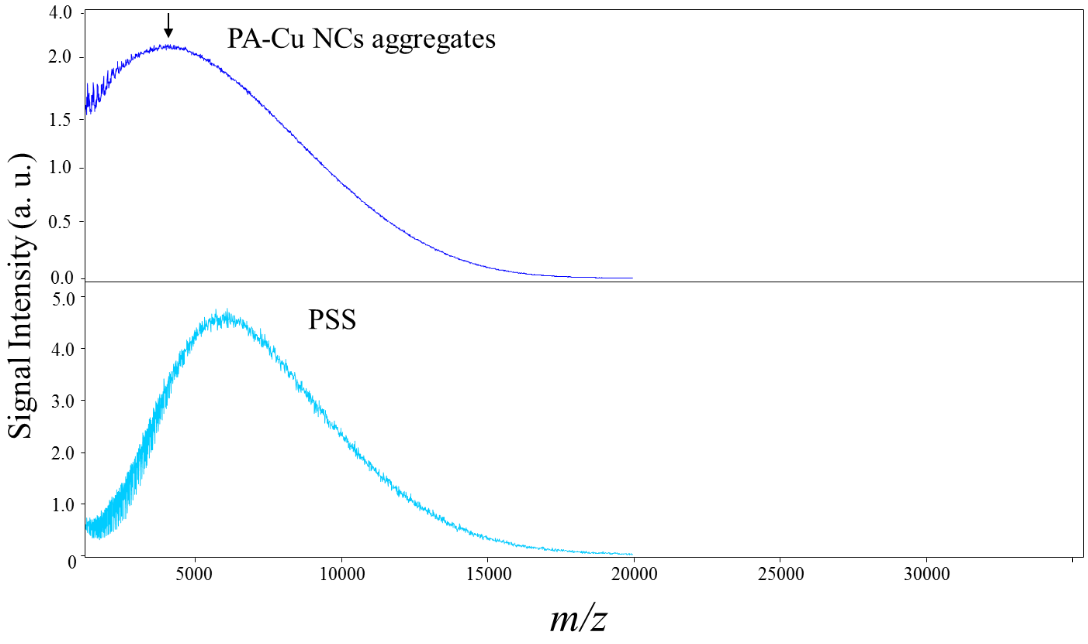

Figure S3. MALDI-TOF-MS spectra of PSS-PA-Cu NC aggregates and PSS.

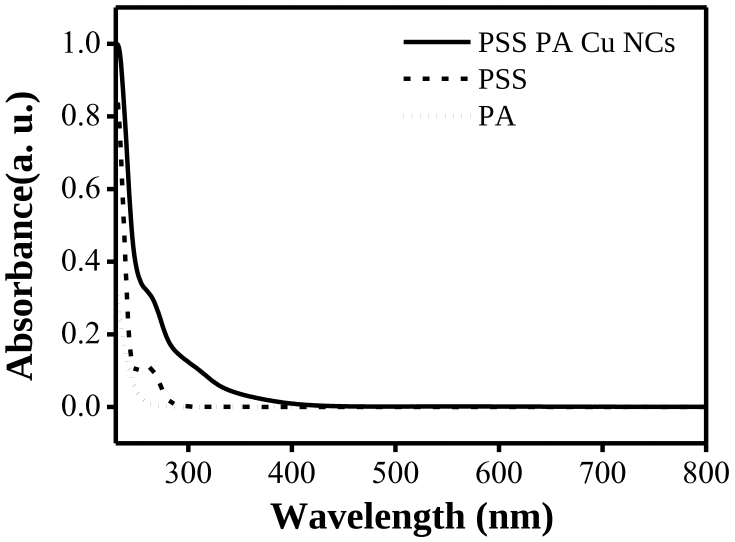

Figure S4. UV-Vis absorption spectra of the PSS-PA-Cu NC aggregates, monomeric PA, and 0.1 wt% PSS aqueous solution.

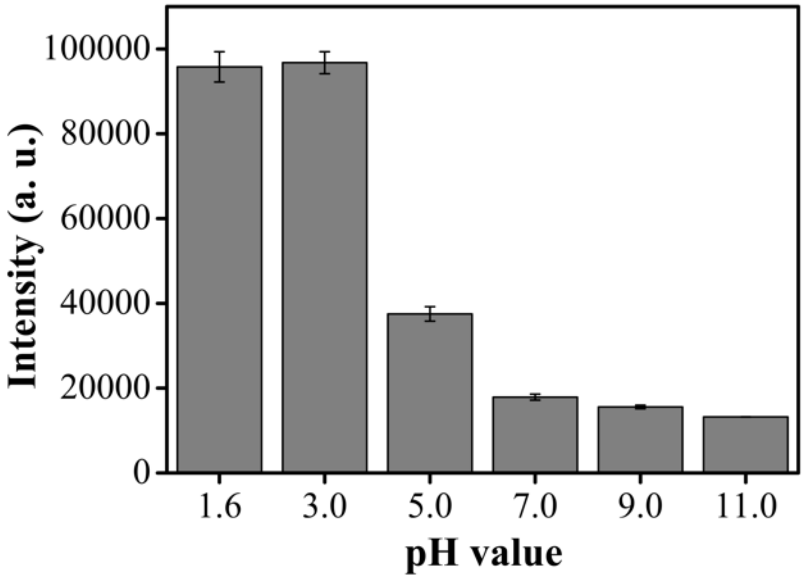

Figure S5. Effects of various pH values (sodium phosphate buffer, 10 mM) on the PL intensity of PSS-PA-Cu NC aggregates (0.05×) (n=3).

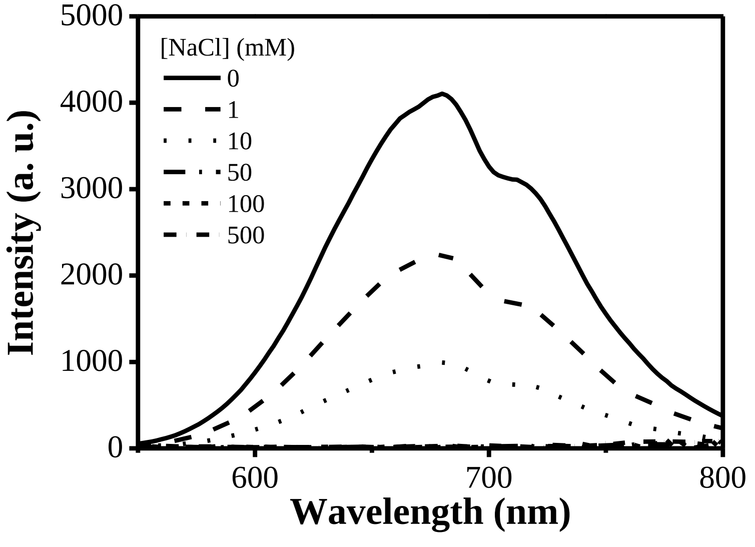

Figure S6. Effects of NaCl concentration on the PL intensity of PSS-PA-Cu NC aggregates (0.05×) prepared in sodium phosphate buffer solutions (pH 3.0, 10 mM). The excitation wavelength was 325 nm.

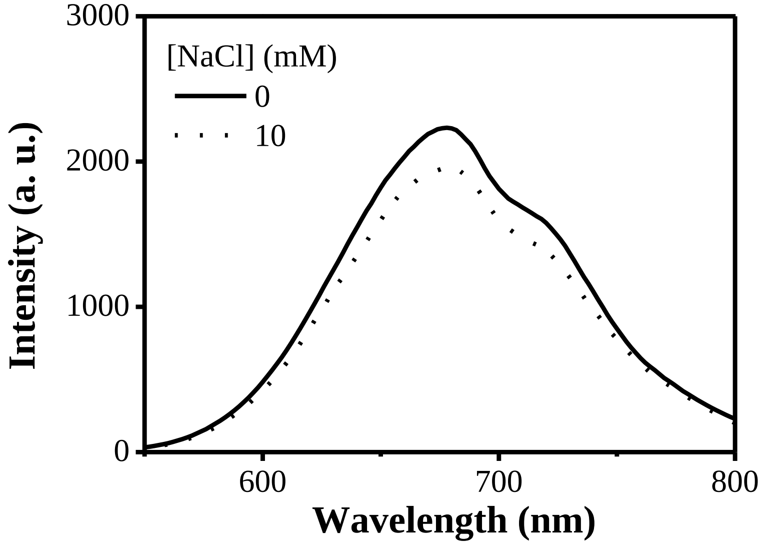

Figure S7. Effects of NaCl on the PL of PA-Cu NC aggregates (0.05×) prepared in sodium phosphate buffer solutions (pH 3.0, 10 mM). The excitation wavelength was 325 nm.

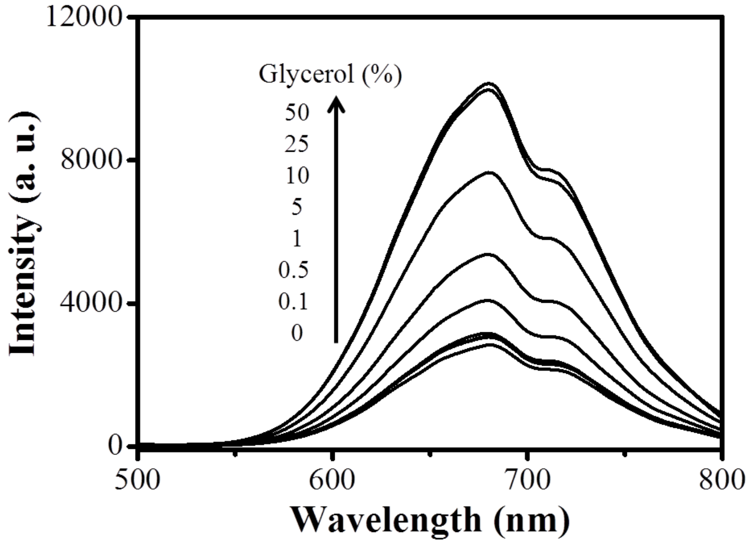

Figure S8. Effects of glycerol on the PL intensity of PSS-PA-Cu NC aggregates (0.05×) in the presence of 50 mM NaCl prepared in sodium phosphate buffer solutions (pH 3.0, 10 mM). The excitation wavelength was 325 nm.

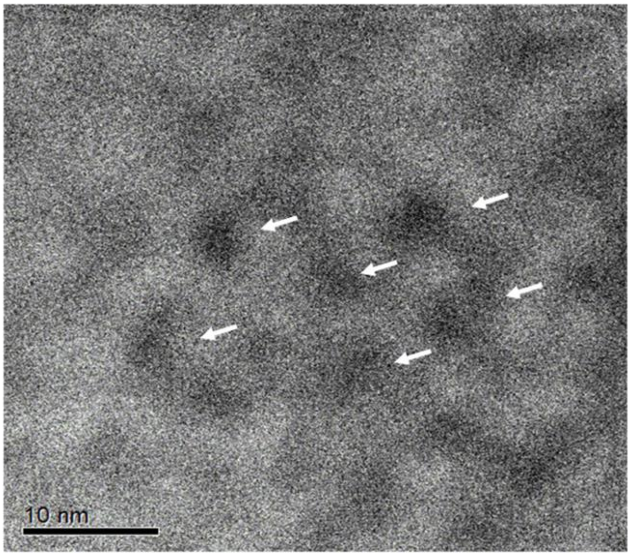

Figure S9. The HR-TEM images of the PSS-PA-Cu NC aggregates in the presence of H_2S (200 μM).

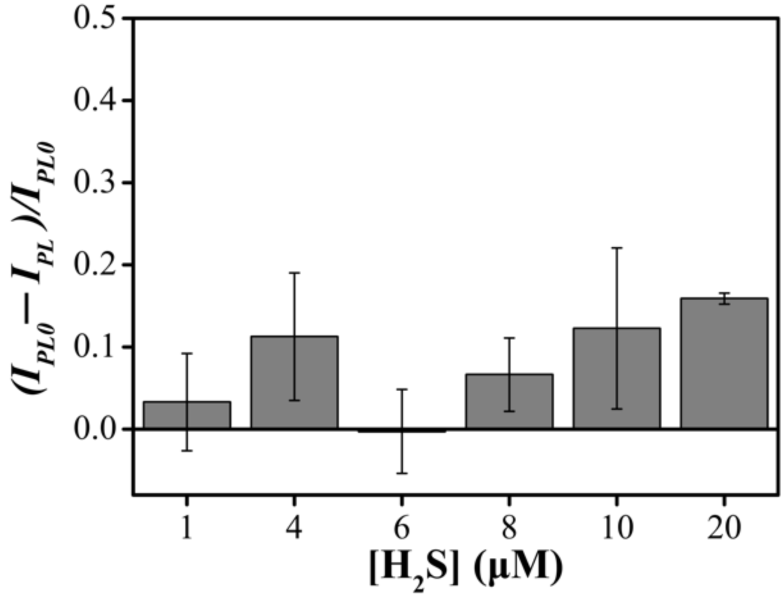

Figure S10. The relative PL intensity of PA-Cu NC aggregates (0.05×) at 665 nm versus different H_2S concentrations. I_{PL0} and I_{PL} are the PL intensities at 665 nm of the PSS-PA-Cu NC aggregates in the absence and presence of H_2S, respectively. The excitation wavelength was 325 nm (n=3).

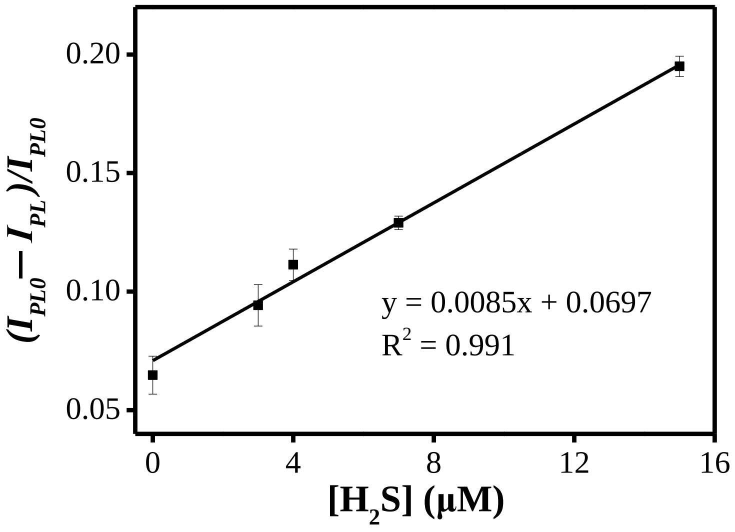

Figure S11. Analyses of representative samples of Beitou hot spring-water samples using PSS-PA-Cu NC/μPAD devices. Diluted (50 fold) hot spring-water samples were spiked with H_2S (0-15 μM) (n=3).
